# Enhancing segmentation fairness through curriculum learning and progressive loss: a centralized and federated perspective on radiograph analysis

**DOI:** 10.3389/frai.2026.1793305

**Published:** 2026-04-17

**Authors:** Ehsan E. Alam, Nickolas Littlefield, Arash Shaban-Nejad, Hamidreza Moradi

**Affiliations:** 1 Department of Computer Science, North Carolina A&T State University, Greensboro, NC, United States; 2 Department of Intelligent Systems, University of Pittsburgh, Pittsburgh, PA, United States; 3 Department of Pediatrics, The University of Tennessee Health Science Center (UTHSC), Memphis, TN, United States

**Keywords:** curriculum learning, fairness, federated learning, hip and knee, progressive loss, segmentation

## Abstract

**Background:**

Bias in medical image segmentation can lead to unequal performance across demographic subgroups, raising concerns about fairness and reliability in clinical AI systems. While deep learning models have achieved high segmentation accuracy, ensuring equitable performance across race and gender remains a significant challenge, particularly in privacy-sensitive healthcare environments.

**Methods:**

This study investigates fairness-aware medical image segmentation for hip and knee radiographs using deep learning models evaluated in both centralized and Federated Learning (FL) settings. We introduce Curriculum Learning (CL) strategies and Progressive Loss (PL) functions to regulate sample difficulty during training. In addition, we propose two novel fairness-oriented federated learning algorithms, Federated Intersection over Union (FedIoU) and Federated Intersection over Union with Outlier Analysis (FedIoUoutlier). Experiments are conducted using multiple segmentation backbones and simulated multi-site data partitions derived from the Osteoarthritis Initiative dataset. Model performance is evaluated using Intersection over Union (IoU), IoU standard deviation, Skewed Error Ratio (SER), and Min-Max Disparity across race and gender subgroups. Statistical significance was verified using paired *t*-tests to compare per-sample IoU performance against baseline configurations.

**Results:**

Across both hip and knee segmentation tasks, curriculum learning and progressive loss strategies consistently improved segmentation accuracy and reduced demographic performance disparities in centralized training. In federated settings, fairness-aware aggregation further enhanced performance. Notably, FedIoUoutlier combined with balanced curriculum learning and tiered progressive loss achieved the highest mean IoU while yielding the lowest SER and Min-Max Disparity, indicating improved fairness without sacrificing accuracy. In several configurations, federated models matched or exceeded the performance of optimized centralized models, with statistically significant improvements in per-sample IoU over baseline configurations.

**Conclusion:**

The results demonstrate that structured training strategies and fairness-aware federated aggregation can jointly improve accuracy, stability, and demographic fairness in medical image segmentation. By integrating curriculum learning, progressive loss, and novel FL algorithms, this work provides a practical pathway toward equitable and privacy-preserving AI systems for medical imaging.

## 1 Introduction

The automated outlining of anatomical features in medical scans plays a vital role in Computer-Assisted Diagnosis (CAD) and treatment strategizing, especially within orthopedic applications concerning the hip and knee (Ghidotti et al., 2024; Ling et al., 2023; Sezer and Sezer, 2020). Accurate segmentation allows medical professionals to measure essential anatomical characteristics, recognize disease-related alterations, and meticulously plan procedures such as Total Hip Arthroplasty (THA) (Gatti and Maly, 2021). The precision of implant sizing and placement depends heavily on accurate anatomical delineation, as achieved through precise segmentation, which in turn plays a critical role in determining surgical success and patient rehabilitation outcomes (Chen et al., 2023; Asseln et al., 2018; Nash and Harris, 2014; Marsilio et al., 2023).

Neural Network (NN) models have recently demonstrated impressive, near-human levels of accuracy in segmenting medical images (Hesamian et al., 2019). Nonetheless, their widespread deployment has raised fairness concerns, as biases related to demographic attributes like race and gender can emerge, potentially widening existing healthcare gaps (Siddique et al., 2021; Nazer et al., 2023). Within medical imaging, biased algorithms risk leading to incorrect diagnoses or unsuitable treatment strategies, which can particularly disadvantage individuals from underrepresented populations (Xu et al., 2023).

Previous studies addressing fairness in medical image segmentation have investigated techniques like balancing datasets, employing adversarial training methods, and creating distinct models for different demographic groups (Littlefield et al., 2023a,b; Correa et al., 2022; Wang and Deng, 2020; Zhang et al., 2018b,a). These methods, however, frequently encounter obstacles, including potentially lowering overall model accuracy, failing to account for the intricate interplay between various attributes, or lacking systematic ways to manage samples of differing difficulty levels (Ma et al., 2024). Consequently, there is an ongoing requirement for approaches that can concurrently boost both model accuracy and fairness.

This study addresses this issue by investigating the application of Curriculum Learning (CL) and Progressive Loss (PL) methodologies. Drawing inspiration from human cognitive development, these techniques organize the training regimen by controlling the complexity of the data shown to the model. This structured approach holds the potential to enhance model robustness, accuracy, and fairness (Bengio et al., 2009; Hacohen and Weinshall, 2019; Weinshall et al., 2018). Our hypothesis is that by methodically steering the learning trajectory from simpler to more intricate segmentation challenges, we can reduce biases while preserving or even enhancing accuracy. Furthermore, we expect CL's structured sample progression and PL's adaptive optimization emphasis interact conceptually to guide the model toward better generalization across all subgroups.

Moreover, acknowledging the importance of data privacy and the inherently distributed nature of healthcare data, our study moves beyond conventional centralized training paradigms. We undertake a thorough analysis using a federated learning framework. Federated learning facilitates collaborative model training across multiple entities (simulated through data acquisition sites) without necessitating the exchange of sensitive raw data. This part of our work evaluates the viability and efficacy of our fairness improvement techniques in a privacy-preserving setting. However, FL may also introduces challenges such as convergence instability, and client drift arising from non-Independent and Identically Distributed (non-IID) data distributions, which our proposed methods aim to address. Beyond privacy preservation, FL introduces several trade-offs that are particularly relevant for medical segmentation. Communication overhead can limit scalability, convergence may become unstable under heterogeneous client distributions, and client drift may emerge when local models specialize too strongly to site-specific data. In addition, privacy-preserving mechanisms such as noisy updates can reduce utility, potentially affecting both segmentation accuracy and the consistency of subgroup performance. These challenges motivate the need for training and aggregation strategies that improve not only global performance but also fairness under decentralized, non-IID conditions.

This study utilizes the Osteoarthritis Initiative (OAI) dataset (Osteoarthritis Initiative, 2024), comprising hip and knee radiographs with manual annotations, to investigate robust and fair image segmentation. Initially, we evaluate several baseline segmentation architectures within a centralized training context to identify the optimal model. Subsequently, leveraging this optimal architecture, we implement and compare various CL methods (e.g., Balanced Curriculum Learning) and PL functions (e.g., Tiered Progressive Loss) to determine the most effective combination for enhancing segmentation performance.

Finally, we conduct a comprehensive FL evaluation in a simulated multi-site setup. This phase involves comparing multiple FL algorithms (e.g., Federated Average, Fair Federated Differential Privacy, Federated Intersection over Union with outlier Analysis), each integrated with local training protocols that incorporate the best-performing CL method and PL function identified in earlier phases. Model performance is rigorously assessed based on accuracy (Intersection over Union), consistency (Standard Deviation), and fairness [Skewed Error Ratio (SER) and Min-Max Disparity] across different race and gender categories. To strengthen the robustness of the findings, we conducted paired *t*-tests on per-sample IoU scores to determine whether the observed improvements over baseline configurations were statistically significant. By preserving patient privacy in a distributed setting, FL allows the development of fair and unbiased models, a crucial requirement often overlooked in centralized approaches to medical data.

The primary contributions of our research are:

- An assessment of how model architecture choices impact fairness and accuracy in centralized segmentation training.
- A demonstration that CL and PL techniques can significantly enhance both accuracy and fairness in centralized segmentation compared to a baseline, with accuracy gains verified through statistical significance testing.
- A detailed comparative analysis of fairness and accuracy within a federated learning context, evaluating various FL algorithms combined with optimized CL and PL strategies using multiple metrics, including SER and Min-Max Disparity.
- The development of novel federated learning configurations and algorithms that achieve state-of-the-art results in fairness and accuracy within a privacy-preserving framework with fairness consideration, in some instances outperforming optimized centralized training approaches.

## 2 Materials and methods

### 2.1 Background and related work

This section delves into three primary areas of related research studies. We begin by discussing existing methodologies aimed at improving model accuracy. Following this, we explore the advancements and challenges surrounding fairness in AI, where data and algorithmic biases can amplify disparities. Our goal is to identify the gaps in integrating both accuracy and fairness in model development, a process that lays the foundation for our study. We then discuss the importance of a Federated focused enhancement, a crucial element that is often missing from centralized studies.

### 2.2 Accuracy focused improvement

In Guan et al. (2018), the authors addressed the challenge of classification accuracy in imbalanced datasets, specifically in the context of diabetic retinopathy detection. Their method incorporated expert knowledge by assigning higher importance to labels considered more reliable, allowing the training process to prioritize those samples. The experimental results demonstrated improvements in overall classification performance. In Ju et al. (2022), the authors addressed the problem of label noise in medical imaging through a curriculum learning framework. Their method assigns weights to samples using normalized uncertainty scores, enabling the model to identify and prioritize more challenging examples during training. Wang et al. (2021a) proposed a multi-task framework for semi-supervised segmentation of MRI images. The model is first trained using labeled data and then progressively incorporates unlabeled samples during training. This strategy improves segmentation performance and is particularly advantageous when labeled data are limited. In Li et al. (2023), the authors focused on improving learning for underrepresented classes by modifying the training strategy. They introduced a task-oriented curriculum learning approach that decomposes the segmentation task into multiple label distributions while calculating class frequency statistics to guide training. Although the studies discussed above (Guan et al., 2018; Wang et al., 2021a; Li et al., 2023; Ju et al., 2022) contribute to improving model accuracy, they do not examine how the proposed approaches affect model fairness.

### 2.3 Fairness focused enhancement

Early studies addressing fairness in medical image analysis primarily concentrated on dataset balancing and augmentation strategies to ensure that models were trained on a diverse and representative set of images. In Seyyed-Kalantari et al. (2020), the authors introduced a framework designed to reduce performance disparities across demographic groups by enforcing similar true positive rates among them. Zhang et al. (2018b) investigated the application of Generative Adversarial Networks (GANs) to expand datasets by generating samples representing underrepresented demographic groups. This approach aimed to enhance dataset diversity and reduce potential model bias during training. In Wang et al. (2021b), the authors examined multi-task learning approaches where demographic attributes are predicted alongside the primary task. Information from the demographic prediction task is then used to guide fairness adjustments in the main prediction task. Zhang et al. (2018a) applied adversarial learning to limit the ability of a discriminator to infer sensitive demographic attributes from the outputs of the predictive model, thereby reducing bias associated with those attributes. Edwards and Storkey (2016) proposed a method based on variational autoencoders to learn feature representations that remain invariant to demographic characteristics. These learned representations were subsequently utilized for downstream predictive modeling tasks. Although considerable progress has been achieved, these approaches generally do not consider the effects of progressively increasing task difficulty or transitioning from simpler samples to more complex ones during training.

### 2.4 Federated learning focused enhancement

The studies discussed above in Sub-sections 2.2 and 2.3 primarily focused on a centralized training paradigm, where the model training process assumes access to the entire dataset locally. While this approach allows for potentially higher model accuracy through centralized optimization, it raises serious privacy concerns, particularly when sensitive medical data must be transferred to a central server. To address this limitation, federated learning emerged as a promising solution by enabling model training across decentralized sites without direct data sharing (McMahan et al., 2017; Yuan and Li, 2022). To strengthen privacy, Dwork et al. (2006) formalized Differential Privacy (DP), introducing mathematically grounded mechanisms to protect individual data contributions by adding calibrated noise. Building on this principle, Ashraf et al. (2022) proposed FedDP, which integrates DP into FL by perturbing local updates before aggregation, offering strong privacy guarantees but often at the cost of reduced model accuracy. Subsequent investigations (Yan et al., 2023; Fang and Ye, 2022) examined this privacy–utility trade-off, showing that excessive noise can substantially degrade performance, particularly for complex segmentation tasks or in highly heterogeneous settings (Kairouz et al., 2021; Truex et al., 2019). More recently, Rafi et al. (2024) highlighted the Privacy-Preserving Fairness Challenge, emphasizing that achieving privacy alone does not ensure equitable performance across demographic groups. These findings underscore the need for methods that jointly promote fairness and privacy within distributed learning. Motivated by these challenges, our work investigates whether advanced training strategies such as Curriculum Learning (CL) and Progressive Loss (PL), combined with fairness-oriented FL algorithms, can mitigate this trade-off and achieve high accuracy and fairness simultaneously within a privacy-preserving distributed framework, a direction largely unexplored in prior centralized research.

### 2.5 Dataset and pre-processing

**Figure 1:**
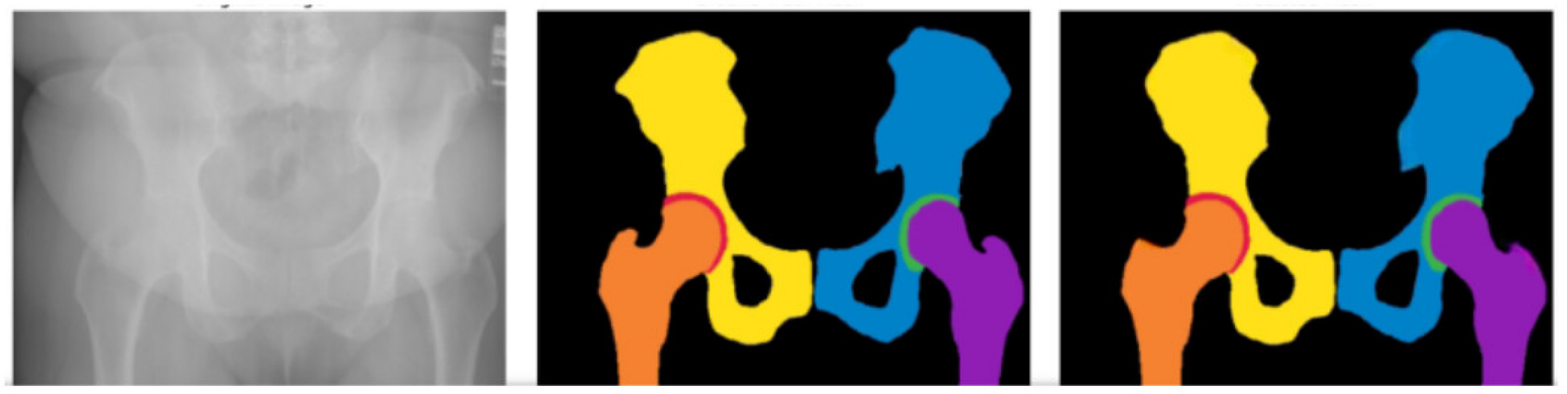
Hip segmentation: original image **(Left)**, Annotated Mask **(Center)**, and Predicted Mask **(Right)** Color mapping: black represents the Background; yellow and blue correspond to the left and right Acetabulum, Ilium, Ischium, and Pubis, respectively; Red and Green indicate joint spaces; Orange and Violet represent the left and right Femur.

**Figure 2:**
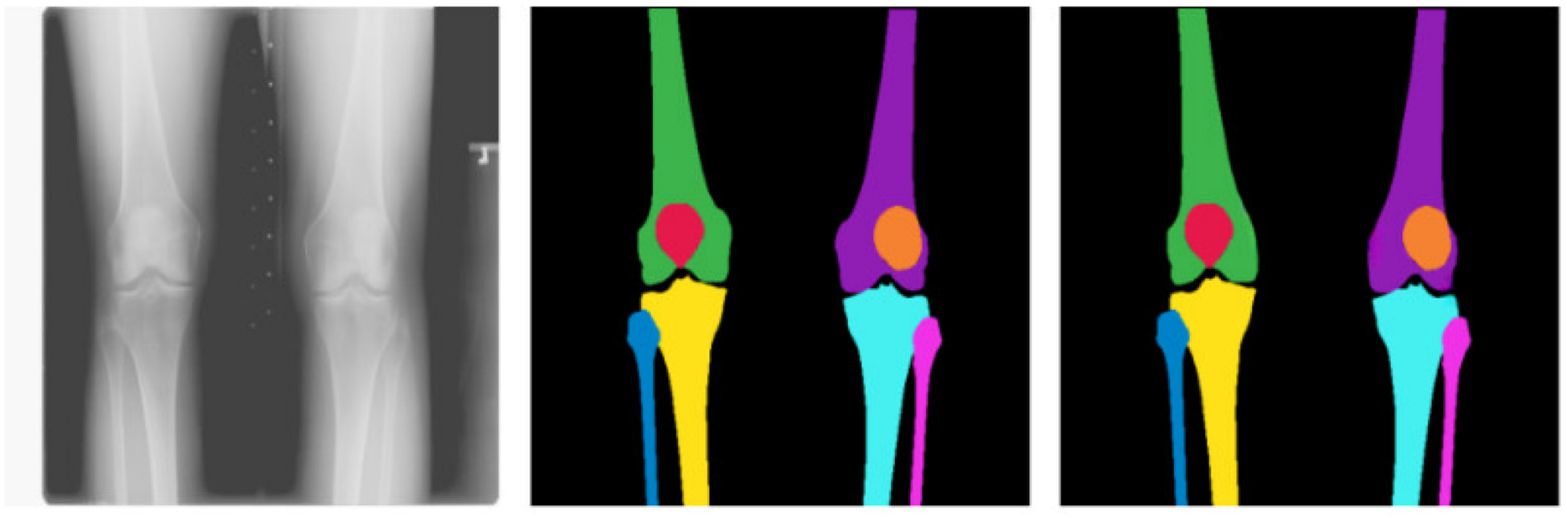
Knee segmentation: original image **(Left)**, annotated mask **(Center)**, and predicted mask **(Right)** Color Mapping: Black represents the Background; Green and Violate represent the Left and Right Femur; Red and Orange represent the Left and Right Patella; Yellow and Cyan represent the Tibia; and Blue and Pink represent the Left and Right Fibula.

This study is based on a retrospective cohort sourced from the Osteoarthritis Initiative (Osteoarthritis Initiative, 2024), with manual annotations (Littlefield et al., 2023b). The randomly chosen cohort consisted of 761 patients who contributed hip radiographs and 707 patients who contributed knee radiographs; each patient provides one X-ray image along with its respective segmentation mask (detailed in Figures 1, 2).

### 2.6 Experimental setup

We used Python 3.10.0 (Python Software Foundation, Wilmington, DE, USA) and PyTorch 1.13.1 [Meta AI (Facebook), Menlo Park, CA, USA] to train the segmentation models in this study. The dataset was divided into training, validation, and testing sets, with 70%, 10%, and 20% of the data randomly selected, respectively. To maintain proportional representation, the demographic distribution (race and gender) are kept consistent across these splits, reflecting the overall dataset distribution.

The hyperparameters for our experiments were selected through an iterative process aimed at optimizing validation accuracy. We tested different epochs, ranging from 25 to 200, and explored both Binary Cross-Entropy (BCE) and Cross-Entropy (CE) losses. The weight decay parameter was adjusted within the range of 1.0 × 10^−3^ to 1.0 × 10^−8^, and the learning rate was varied between 0.01 and 0.0001 to ensure stable and efficient model convergence. The Adam optimizer, which provided the most reliable results, was used consistently across all models and configurations.

### 2.7 Baseline architectures

Our investigation involved a variety of segmentation models with differing architectures and parameter sizes to determine their effectiveness in enhancing fairness and accuracy. The models studied include U-Net (Ronneberger et al., 2015), LinkNet (Chaurasia and Culurciello, 2017), MANet (Xu et al., 2021), PAN (Khosravan et al., 2019), and PSPNet (Zhu et al., 2021). Each of these models has a unique structure, offering a way to understand how varying levels of design intricacy influence medical image segmentation performance.

Note that the architecture identified as optimal, achieving the highest equilibrium between accuracy and fairness, will then become the baseline for evaluating several training methods discussed in the remainder of this section.

### 2.8 Baseline modeling approaches

The subsequent part outlines various training methodologies, each formulated to yield a distinct variant of a baseline architecture being studied.

Standard Baseline (Base): This model is trained on a random reordering of the training data before the start of every epoch. Such randomization ensures the model processes a diverse sequence of samples during each training cycle and that the learning process is not skewed by any particular initial arrangement of the data.

Randomized Interleaving (RI): For this model, an alternating sampling method was utilized to promote varied exposure to different demographic cross-sections, with a specific emphasis on race and gender. Throughout each training epoch, one sample was chosen at random from every race-gender classification, establishing a cycle that continues until all samples in the dataset have been processed. This technique generates a data sequence where examples from varying demographic cohorts (White Males, Black Females, Black Males, and White Females) are interwoven during the training. Our aim is to evaluate how the methodical alternation of diverse demographic examples, based on race and gender, affects segmentation performance.

Demographically-Tailored Models (DS): In this training strategy, distinct models are individually trained for each specific race-gender pairing, which leads to four separate models: White Male (WM), Black Female (BF), Black Male (BM), and White Female (WF). Such a method enables us to investigate the impact of creating customized models for each of these demographic segments. Every model is subjected to its own training process and hyperparameter tuning, guaranteeing a specialized approach for each distinct group. By concentrating on models tailored to specific demographics, our objective is to determine how these customized models influence both the accuracy and fairness of the segmentation.

### 2.9 Curriculum learning based approaches

Self-Paced Curriculum Learning (SCL): Typically, Curriculum Learning methodologies are designed to incrementally raise the complexity of the learning task. In the specific application of the SCL model, the initial phase of training start with a randomized ordering of the training data. However, from the second epoch onward, the training samples are reordered based on the Intersection over Union (IoU) scores achieved by the model from the immediately preceding epoch. This process arranges all training data from the highest IoU scores to the lowest, thereby creating a progression from less difficult to more difficult examples. This technique employs the IoU score as a proxy for how challenging a sample is from the model's viewpoint.

Furthermore, we incorporated a demographic interleaving technique to ensure the model consistently processed data from diverse demographic cohorts throughout its training. This involves organizing the dataset in an alternating sequence of different race-gender categories. By giving priority to samples that achieve the highest IoU scores within each demographic group, our goal is to expose the model comprehensively to all race-gender combinations while simultaneously increasing the overall difficulty of the samples it encounters.

Teacher-Student Curriculum Learning (TCL): This technique diverges from the SCL strategy, which relies on the current model's epoch-by-epoch performance to determine sample prioritization for the subsequent epoch. Instead, this method harnesses a teacher model's in-depth understanding of sample difficulty. Specifically, we employed the baseline model, which had been extensively trained to its optimal performance level, to act as a proficient teacher guiding the student model.

Throughout the training, following each epoch, IoU scores are computed for every sample using both the teacher and the student models. The discrepancy between these two performance scores is then calculated. This difference value serves as the indicator of a sample's difficulty and is used to arrange the training samples for the next epoch. Minor differences in performance suggest the student model is approaching the highest attainable accuracy for those samples, classifying them as easier. In contrast, larger differences indicate that the samples are more difficult. Therefore, this approach means the student model does not depend on the teacher's accuracy as a fixed benchmark of difficulty; rather, it considers the continuously updated and evolving performance gap between the two.

Consistent with other approaches, we also incorporated a demographic interleaving method to ensure the model was regularly exposed to diverse demographic cohorts during the training process.

Balanced Curriculum Learning (BCL): Differing from the previously mentioned curriculum learning methods, BCL does not leverage the entire training dataset during every epoch. The BCL model initiates its training with a randomly selected, demographically balanced subset, which constitutes 20% of the total training data. Subsequently, the size of this training dataset is gradually enlarged with each passing epoch, with the goal of incorporating the full training dataset by the time the final training epoch is reached. Furthermore, when introducing new samples from the remaining training pool, the model first calculates the IoU score for these unseen instances. These IoU scores are then used to identify and select the less complex samples for inclusion in the next training cycle. Once the dataset for the upcoming epoch is assembled, these samples are re-sorted based on their IoU scores, from highest to lowest, ensuring that tasks perceived as easier are prioritized. This strategy facilitates a progressive exposure of the model to samples of increasing difficulty across epochs, allowing it to thoroughly learn from simpler examples before being presented with more challenging ones in later stages. Similar to the other models, a demographic interleaving approach was also integrated to ensure consistent exposure to diverse demographic groups throughout the training.

It should be noted that upon identifying the most effective curriculum learning technique, this method will provide the groundwork for the evaluation of various progressive loss functions, which are elaborated upon in the subsequent portion of this section.

### 2.10 Progressive loss based approaches

This part discusses progressive loss functions engineered to dynamically steer the training process. They enable models to effectively transition their learning from less challenging to more difficult instances as training progresses, by leveraging the varying difficulty levels of samples and gradually shifting the instructional focus.

Selective Progressive Loss (SPL): This particular loss function initially directs the training process to give more weight to samples that the model predicts with higher accuracy. This allows the optimization to be more significantly shaped by the gradients originating from these simpler instances. As the training progresses, the contribution of these easier samples diminishes progressively within each epoch, eventually leading to an inversion of their influence. Consequently, this indirectly amplifies the impact of more difficult samples.

The SPL loss function is formulated as follows:


$$\begin{array}{l} L_{\text{SPL}}=L_{\text{standard}}+w\times \overset{N}{\underset{i=1}{\sum }}\max\left(\Delta P_{i},0\right) \end{array}$$ (1)

In this Equation 1, $\overset{N}{\underset{i=1}{\sum }}$ sums the contributions from all *N* training samples. The term Δ*P*_*i*_ signifies the performance difference for the *i*-th sample compared to the average performance of all samples within that epoch (specifically, *IoU*_*i*_ − *IoU*_avg. epoch_). The variable *w* acts as an adjustable parameter. This parameter *w* is dynamically modified during the training period, beginning at a value of +α and systematically decreasing to −α by the conclusion of the training.

Tiered Progressive Loss (TPL): Analogous to the SPL method, this loss function initially emphasizes samples that the model predicts with higher correctness during the early phases of training. As the training continues, however, the sway of these simpler examples gradually diminishes with each epoch, and, in due course, more complex samples start to command greater attention.

By the later stages of training, the learning process predominantly centers on the more challenging samples. This focus is particularly directed toward those instances that are still not performing as well as the average of all training examples. Consequently, these difficult, underperforming samples exert a more substantial shaping force on the training dynamics as it nears completion.


$$\begin{array}{l} L_{TPL}=L_{standard}+\overset{N}{\underset{i=1}{\sum }}\max\left(w\times \Delta P_{i},0\right) \end{array}$$ (2)

In Equation 2, *w* serves as an adjustable hyperparameter that transitions from an initial value of +α and is progressively reduced to -α by the conclusion of the training period. As it takes on negative values, samples where the model is performing poorly (underperforming examples) exert a more significant influence on the loss calculation, prompting the training process to adapt and effectively manage these challenging instances.

After identifying the most effective CL methods and PL functions, our focus will shift from centralized learning to distributed learning within the FL framework, with a strong emphasis on fairness considerations. Although CL regulates the order of sample exposure during local training and PL adjusts optimization emphasis through adaptive loss weighting, the proposed FL aggregation strategies operate at the global level by determining how local client updates contribute to the shared global model. Conceptually, these components are complementary rather than redundant. CL structures the sequence of local sample exposure, allowing the model to establish stable representations before confronting harder cases. PL further reshapes the optimization trajectory by progressively increasing attention to underperforming examples. Federated-based aggregation then operates at the server level, prioritizing client updates that reflect stronger local segmentation quality. Together, these mechanisms act at different stages of learning and are expected to interact synergistically by improving local robustness before global aggregation.

### 2.11 Federated learning based approaches

Acknowledging the critical importance of data privacy and the inherently distributed nature of medical data, we further investigated federated learning. This enables collaborative model training across multiple decentralized clients (e.g., hospitals or acquisition sites) without sharing raw patient data, thereby offering a privacy-preserving alternative to traditional centralized training (McMahan et al., 2017; Kairouz et al., 2021). In this study, we simulate a multi-site FL environment to evaluate the efficacy of our proposed fairness enhancing strategies. This setting not only preserves data confidentiality but also allows us to examine how fairness oriented methods perform when demographic and site specific imbalances are distributed across clients, offering a more realistic and challenging testbed for equitable model development.

For the FL experiments, the data was partitioned to simulate distinct acquisition sites, reflecting a realistic scenario where data is siloed. Each site (client) possesses its own local dataset. Models are trained locally per global communication round. The objective is to assess how different FL algorithms, combined with our fairness focused training configurations, perform in terms of accuracy and fairness. Although this site-based partitioning provides a practical proxy for decentralized learning, the federated setting remains a simulation derived from a single dataset rather than a real multi-institutional deployment. This design allows us to study the fairness and performance behavior of the proposed algorithms under privacy-preserving conditions, while acknowledging that further validation across independent institutions would be required for real-world deployment.

We investigated a comprehensive suite of FL algorithms to understand their impact on model performance and fairness:

Federated Averaging (FedAvg): As the foundational algorithm in FL, Federated Averaging (FedAvg) employs an iterative, collaborative training process. The cycle commences with a central server distributing an initial global model to all participating clients. Each client then trains this model using its own local, private data to produce an updated set of model parameters. These locally updated models are subsequently sent back to the central server for aggregation. The server combines them to form a new, improved global model by computing a weighted average of the client models. The influence of each client is proportional to the size of its local dataset, thereby giving more weight to clients with more data. While notable for its simplicity and efficiency, FedAvg's performance can be hindered by significant statistical heterogeneity across clients, making it an essential baseline for evaluating FL methods in this study.


$$\begin{array}{l} \theta_{t+1}=\overset{K}{\underset{k=1}{\sum }}\frac{n_{k}}{n}\theta_{t}^{k} \end{array}$$ (3)

In this Equation 3, θ_*t*+1_ represents the parameters of the aggregated global model for the next training round, *t* + 1. This is computed by adding and scaling the contributions from all *K* clients. The term $\theta_{t}^{k}$ denotes the model parameters submitted by client *k* after its local training phase *t*. The influence of each client's update is scaled by the factor $\frac{n_{k}}{n}$, representing the proportion of data samples held by client *k* (*n*_*k*_) relative to the total number of samples across all clients (*n*). This weighting scheme ensures that clients with larger datasets contribute more significantly to the updated global model.

Federated Proximal (FedProx): This algorithm extends FedAvg by modifying the local objective function with a proximal term. Each client *k* minimizes


$$\begin{array}{l} \underset{\theta }{\min}f_{k}\left(\theta \right)+\frac{\mu }{2}\|\theta -\theta_{t}{\|}^{2}, \end{array}$$ (4)

In this Equation 4, where *f*_*k*_(θ) is the client's local loss function and the proximal term $\frac{\mu }{2}\|\theta -\theta_{t}{\|}^{2}$ penalizes large deviations from the global parameters θ_*t*_. This ensures that local updates remain closer to the global model, improving training stability. After local training, the server aggregates the client updates using the same weighted averaging rule as in FedAvg.

Federated Differential Privacy (FedDP): FedDP enhances FedAvg by incorporating a rigorous privacy guarantee known as differential privacy. To ensure that the central server cannot infer sensitive information about any single participant from a client's update, each client adds a controlled amount of random noise to its model parameters before transmitting them. This noise perturbs the update just enough to protect individual data, while still allowing the global aggregation to capture meaningful patterns. In our study, we varied the noise standard deviation from 0.1 to 0.00001 in order to explore the trade-off between privacy preservation and model performance.


$$\begin{array}{l} {\tilde{\theta }}_{t}^{k}=\theta_{t}^{k}+N\left(0,\sigma^{2}\right), \end{array}$$ (5)

In Equation 5 where $\theta_{t}^{k}$ denotes the local model parameters of client *k* at round *t*, and ${\tilde{\theta }}_{t}^{k}$ represents the privatized version that is sent to the server. The added noise $N\left(0,\sigma^{2}I\right)$ is sampled from a Gaussian distribution with zero mean and variance σ^2^, applied independently across all parameters. The parameter σ controls the strength of the privacy guarantee: larger values of σ provide stronger privacy by increasing uncertainty about individual contributions, but can also degrade model accuracy, whereas smaller values preserve more utility at the expense of weaker privacy guarantees. After privatization, the server aggregates the noisy updates using the same weighted averaging rule as in FedAvg.

Federated Differential Privacy with Fairness (FedDPfair): In this algorithm we extended the FedDP by integrating a novel fairness-aware aggregation mechanism. Similar to FedDP, each client perturbs its local update by adding Gaussian noise to guarantee differential privacy. The key difference lies in the aggregation stage: instead of weighting clients solely by dataset size, FedDPfair adjusts the contribution of each client according to its fairness performance. Specifically, the weight assigned to client *k* is inversely proportional to its Skewed Error Ratio (SER), as defined in Section 2.12 (Wang and Deng, 2020), such that models exhibiting lower SER (i.e., more equitable performance across groups) have greater influence on the global model.


$$\begin{array}{l} \theta_{t+1}=\overset{K}{\underset{k=1}{\sum }}\frac{w_{k}}{\sum_{j=1}^{K}w_{j}}{\tilde{\theta }}_{t}^{k},\;\text{with }w_{k}=\frac{1}{{\text{SER}}_{k}}, \end{array}$$ (6)

In Equation 6, where ${\tilde{\theta }}_{t}^{k}$ denotes the noise-perturbed parameters of client *k*, and SER_*k*_ is the Skewed Error Ratio computed from that client's model. The weight *w*_*k*_ = 1/SER_*k*_ ensures that clients with smaller SER values, indicating fairer performance across demographic subgroups, receive proportionally higher importance in the aggregation process. The normalization factor $\overset{K}{\underset{j=1}{\sum }}w_{j}$ guarantees that the weights form a valid convex combination, so that the global update θ_*t*+1_ remains within the parameter space. This fairness-aware reweighting mechanism balances privacy preservation with equitable model performance across clients.

Federated Intersection over Union (FedIoU): This novel FL algorithm, designed specifically for segmentation tasks, improves server-side aggregation by weighting each client's contribution according to its local Intersection over Union (IoU) score. Analogous to FedDPfair, but using IoU instead of 1/SER, this approach assigns greater influence to clients that achieve stronger segmentation performance on their local data (see Section 2.12 for the formal definition of IoU). The shift from dataset-size weighting (FedAvg) to IoU-based weighting emphasizes segmentation quality during aggregation rather than client sample count alone. In principle, this allows the global model to place greater importance on high-quality local updates.


$$\begin{array}{l} \theta_{t+1}=\overset{K}{\underset{k=1}{\sum }}\frac{s_{k}}{\sum_{j=1}^{K}s_{j}}\theta_{t}^{k},\;\text{with }s_{k}={\text{IoU}}_{k}, \end{array}$$ (7)

In Equation 7, where $\theta_{t}^{k}$ denotes the parameters of client *k* after local training, and IoU_*k*_ is the segmentation score computed from that client's model. The term *s*_*k*_ thus acts as a performance-based weight, and the denominator $\overset{K}{\underset{j=1}{\sum }}s_{j}$ represents the total weight across all clients, ensuring normalization. In this way, clients with higher IoU values contribute proportionally more to the global update, encouraging the aggregation to emphasize well-performing models and thereby improving overall segmentation accuracy.

Federated Intersection over Union with Outlier Analysis (FedIoUoutlier): This method extends FedIoU by incorporating a statistical outlier detection mechanism to improve robustness against anomalous updates. At the start of each aggregation round, local IoU scores are collected and analyzed using the Interquartile Range (IQR) method. Clients with IoU scores falling below the lower threshold, defined as *Q1* − 1.5 × IQR, are treated as outliers and excluded from the aggregation. This exclusion prevents low-quality or unstable models from disproportionately degrading the global model. FedIoUoutlier acts as a robustness-oriented aggregation strategy. By statistically pruning anomalous client updates through IQR analysis, this method can help mitigate the effects of client drift and convergence instability that often arise in non-IID medical data. This filtering mechanism reduces the influence of underperforming or unstable local updates, thereby improving the robustness of the aggregated global model.


$$\begin{array}{l} \;\theta_{t+1}=\sum_{k\in A}\frac{s_{k}}{\sum_{j\in A}s_{j}}\theta_{t}^{k},\\ \text{with }A=\{k:{\text{IoU}}_{k}\geq \text{Q1}-1.5\times \text{IQR}\}. \end{array}$$ (8)

In Equation 8, where A is the set of non-outlier clients, $\theta_{t}^{k}$ denotes the parameters of client *k* after local training, and *s*_*k*_ = IoU_*k*_ represents its segmentation score. The denominator $\underset{j\in A}{\sum }s_{j}$ represents the total weight of all retained clients, ensuring that the normalized coefficients sum to one and the global update θ_*t*+1_ remains a convex combination of valid models. By filtering out underperforming or anomalous clients, FedIoUoutlier emphasizes high-quality contributions, thereby improving both the stability and accuracy of the global model. This design is particularly relevant when combined with BCL and TPL. Because BCL promotes more balanced and difficulty-aware local learning, and TPL shifts optimization toward harder samples later in training, the resulting client models are expected to be both stronger and more stable before aggregation. Under such conditions, IoU-based aggregation can more effectively identify informative client updates, while the outlier filtering mechanism reduces the risk that unstable local models dominate the global update.

### 2.12 Evaluation metrics

Intersection over Union (IoU): As a pixel-level classification task, image segmentation is often evaluated with the IoU score. IoU gauges the similarity between AI-generated and gold-standard segmentation masks by calculating the ratio of their pixel intersection to their union, a process detailed in the subsequent Equation 9.


$$\begin{array}{l} \text{IoU}=\frac{{\text{Intersection}}_{\text{predicted}\&\text{actual}}}{{\text{Union}}_{\text{predicted}\&\text{actual}}} \end{array}$$ (9)

Pixels accurately identified by the model form the intersection, while the union encompasses the total area of pixels from both the predicted output and the true annotation. The IoU score ranges from 0 (no overlap) to 1 (perfect overlap), with higher scores signifying greater agreement between the prediction and the actual label, thereby reflecting better accuracy.

Standard Deviation (SD): Standard deviation (SD) is a widely used statistical tool that measures how much a set of data points spread out from their average (mean) value. It helps understand the degree to which individual data points vary from this central average, thereby indicating the overall variability within the dataset. In this context, calculating the standard deviation for the IoU scores of a specific protected group reveals the spread in segmentation accuracy around the group's average IoU. This provides insights into the consistency and dependability of the model's performance for that particular group.

Skewed Error Ratio (SER): For fairness assessment, we employed the Skewed Error Ratio (SER) (Wang and Deng, 2020), which analyzes disparities in prediction errors across diverse groups. The SER, as defined in Equation 10, is the ratio of the maximum to the minimum error rates among the protected groups (g). Higher than 1 SER values are indicative of more substantial bias, whereas values closer to 1 imply less bias.


$$\begin{array}{l} {\text{SER}}_{g}=\frac{\max_{g}\left(1-{\text{IoU}}_{g}\right)}{\min_{g}\left(1-{\text{IoU}}_{g}\right)} \end{array}$$ (10)

Employing the SER metric allows us to understand the magnitude of differences and disproportions in the model's predictive accuracy when compared across various protected demographic groups.

Min-Max Disparity (MMD): To complement the SER and provide a more intuitive measure of absolute performance gaps, we introduce Min-Max Disparity (MMD). While SER measures the ratio of errors, MMD quantifies the absolute range of IoU scores across demographic subgroups. It is defined as the difference between the maximum and minimum mean IoU achieved among the protected groups (*g*) shown in Equation 11:


$$\begin{array}{l} MMD_{g}=\underset{g}{\max}\left(IoU_{g}\right)-\underset{g}{\min}\left(IoU_{g}\right) \end{array}$$ (11)

Lower MMD values signify that the model maintains consistent segmentation quality across all demographic categories, ensuring that no single subgroup is significantly disadvantaged.

Statistical Significance Analysis: To ensure that the performance variations observed across different experimental configurations represent mathematically robust improvements rather than stochastic noise, we performed two-tailed paired *t*-tests on the per-sample IoU (P-IoU) scores (Manfei et al., 2017). Because the centralized CL, PL, and FL experiments involve different comparison families, the paired *t*-tests were conducted against the most relevant matched reference configuration within each experimental setting. The exact reference used for each table is specified in the Results section. A *p*-value threshold of *p* < 0.05 was utilized to establish statistical significance. The interpretation of the observed *p*-values followed the standard evidence-strength criteria summarized in Table 1.

**Table 1:** Interpretation of statistical significance via *p*-values.

*p*-value range	Strength of evidence	Significance level
*p* < 0.05	Strong evidence	Statistically significant
*p* ≥ 0.05	Insufficient evidence	Not significant

## 3 Results

### 3.1 Data distribution and base model

The demographic composition of our dataset highlights clear imbalances in terms of gender and race distribution. Both the hip (Table 2) and knee (Table 3) datasets exhibit uneven representation, with the hip data including 324 males and 437 females, and the knee data containing 280 males and 427 females. In terms of race, the hip dataset consists of 443 White and 318 Black individuals, while the knee dataset comprises 447 White and 260 Black participants.

**Table 2:** Demographic counts in the hip dataset.

Demographic	Gender	Race	Race-gender
Male	324		
Female	437		
White or Caucasian		443	
Black or AA^*^		318	
Male-Black (MB)			94
Male-White (MW)			230
Female-Black (FB)			224
Female-White (FW)			213

AA^*^, African American.

**Table 3:** Demographic counts in the knee dataset.

Demographic	Gender	Race	Race-gender
Male	280		
Female	427		
White or Caucasian		447	
Black or AA^*^		260	
Male-Black (MB)			86
Male-White (MW)			194
Female-Black (FB)			174
Female-White (FW)			253

AA^*^, African American.

These demographic discrepancies underscore the importance of mitigating potential biases in our segmentation models. Unequal representation across gender and race can negatively impact model performance and fairness, especially for underrepresented groups. Addressing these disparities through fairness-aware training strategies is essential to develop equitable, reliable, and generalizable models.

In this study, we assessed multiple segmentation models to determine the most effective architecture for further analysis. The evaluation was based on metrics including Average IoU, IoU standard deviation (SD), mean Skewed Error Ratio (SER), and Min-Max Disparity (MMD), as summarized in Tables 4, 5. Since this initial phase serves as a structural benchmark to identify the optimal backbone architecture rather than a hypothesis-driven comparison of new methodologies, per-sample statistical significance tests were deferred to the subsequent evaluation of mitigation strategies.

**Table 4:** Segmentation models performance for hip dataset with disparity metrics.

Model	Mean IoU	IoU SD	R&G^*^mean SER	MMD
LinkNet	0.8654	0.0308	1.4979	0.0774
MANet	0.9249	0.0112	1.3310	0.0278
PAN	0.8511	0.0220	1.3024	0.0577
PSPNet	**0.9312**	0.0190	1.6623	0.0528
U-Net	0.9201	**0.0027**	**1.0655**	**0.0067**

R&G^*^, Race and Gender. The bold values indicate the best-performing results for each metric. Specifically, higher values are highlighted for performance metrics such as IoU, while lower values are highlighted for disparity-related metrics like as SER and Min-Max Disparity.

**Table 5:** Segmentation models performance for knee dataset with disparity metrics.

Model	Mean IoU	IoU SD	R&G^*^mean SER	MMD
LinkNet	0.7956	0.0246	**1.1084**	0.0604
MANet	0.8897	0.0047	1.2422	0.0242
PAN	**0.9374**	0.0081	1.2476	0.0226
PSPNet	0.9226	0.0134	1.1727	0.0338
U-Net	0.9241	**0.0040**	1.1096	**0.0093**

R&G^*^, Race and Gender. The bold values indicate the best-performing results for each metric. Specifically, higher values are highlighted for performance metrics such as IoU, while lower values are highlighted for disparity-related metrics like as SER and Min-Max Disparity.

For hip segmentation (Table 4), U-Net exhibited strong overall performance. It achieved a mean IoU of 0.9201, slightly below PSPNet's 0.9312, but surpassed PSPNet significantly in terms of stability, demonstrated by the lowest IoU SD of 0.0027. Additionally, U-Net had the lowest mean SER at 1.0655 and the minimum absolute MMD of 0.0067, which is substantially lower than models like LinkNet (MMD: 0.0774) or PSPNet (MMD: 0.0528). These findings underscore U-Net's reliability in providing equitable performance by narrowing both the relative error ratio and the absolute accuracy gap between demographic groups.

Similarly, for knee segmentation (Table 5), U-Net performed exceptionally with a mean IoU of 0.9241 and an IoU SD of 0.0040. Although PAN achieved a higher mean IoU of 0.9374, its greater IoU SD (0.0081) and elevated mean SER (1.2476) suggest reduced stability relative to U-Net. U-Net maintained the lowest absolute disparity with an MMD of 0.0093, whereas PAN exhibited more than double the performance gap (MMD: 0.0226). This further emphasizes U-Net's capability to balance accuracy and fairness across diverse populations.

Interestingly, model size alone did not dictate performance. Larger models such as PSPNet (49.7 million parameters) and PAN (50 million parameters) yielded slightly higher accuracy on hip and knee datasets respectively but did not consistently maintain performance across all metrics. Furthermore, these more complex models demonstrated greater bias according to the race and gender mean SER and absolute MMD. This highlights the need to balance complexity, accuracy, and fairness. Despite having fewer parameters (11.7 million), U-Net delivered superior consistency and fairness, indicating that architectural design and generalization ability to varied demographic groups are more influential than parameter count in segmentation tasks. Consequently, U-Net was selected as the optimal baseline architecture for evaluating the Curriculum Learning, Progressive Loss, and Federated Learning strategies.

### 3.2 Curriculum learning performance with U-Net

We employed U-Net as the baseline model to assess various curriculum learning strategies aimed at enhancing accuracy and fairness for both hip and knee segmentation tasks. The outcomes are summarized in Tables 6, 7. To ensure a rigorous evaluation, each strategy was statistically compared against the Standard Baseline (Base) using paired *t*-tests to identify meaningful performance gains.

**Table 6:** Curriculum learning and hip segmentation with statistical and disparity metrics.

Model	Mean IoU	IoU SD	R&G mean SER	MMD	P-IoU
Baseline Model (Base)	0.9201	0.0027	1.0655	0.0067	–
Random Interleaved (RI)	0.9215	0.0103	1.2989	0.0233	0.22
Demographic-Specific (DS)	0.6586	0.0125	1.1450	0.0457	**<0.05**
Self-Paced (SCL)	0.9147	0.0029	1.0284	0.0076	**<0.05**
Teacher-Student (TCL)	0.9271	0.0026	1.0432	0.0073	**<0.05**
Balanced (BCL)	**0.9664**	**0.0002**	**1.0120**	**0.0004**	**<0.05**

The bold values indicate the best-performing results for each metric. Specifically, higher values are highlighted for performance metrics such as IoU, while lower values are highlighted for disparity-related metrics like as SER and Min-Max Disparity.

**Table 7:** Curriculum learning and knee segmentation with statistical and disparity metrics.

Model	Mean IoU	IoU SD	R&G mean SER	MMD	P-IoU
Baseline Model (Base)	0.9241	0.0040	1.1096	0.0093	–
Random Interleaved (RI)	0.9214	0.0110	1.1938	0.0300	0.35
Demographic-Specific (DS)	0.8736	0.0104	1.1863	0.0216	**<0.05**
Self-Paced (SCL)	0.9453	0.0028	1.0817	0.0077	**<0.05**
Teacher-Student (TCL)	0.9394	0.0025	1.0705	0.0068	**<0.05**
Balanced (BCL)	**0.9661**	**0.0002**	**1.0088**	**0.0005**	**<0.05**

The bold values indicate the best-performing results for each metric. Specifically, higher values are highlighted for performance metrics such as IoU, while lower values are highlighted for disparity-related metrics like as SER and Min-Max Disparity.

For hip segmentation (Table 6), the Balanced Curriculum Learning (BCL) approach demonstrated superior performance compared to other methods, achieving the highest mean IoU of 0.9664, the lowest variation (IoU SD: 0.0002), and the smallest mean SER value of 1.0120. Additionally, BCL achieved Min-Max Disparity (MMD) of 0.0004, which was found to be highly significant compared to baseline. The BCL method incrementally introduced more difficult samples during training, promoting fairness and consistent results across demographic groups. By comparison, the Baseline Model recorded a mean IoU of 0.9201 but showed higher bias, as indicated by a race and gender mean SER of 1.0655 and an absolute MMD of 0.0067. In contrast, the Random Interleaved (RI) approach failed to achieve statistical significance. Despite a marginal increase in mean IoU, RI actually exacerbated demographic bias, as evidenced by an increased SER of 1.2989 and a higher MMD of 0.0233, identifying that simple demographic shuffling without a difficulty curriculum is insufficient.

For knee segmentation (Table 7), a similar pattern was noted. The Balanced Curriculum Learning (BCL) model again delivered the best performance, with a mean IoU of 0.9661, an IoU standard deviation of 0.0002, and a mean SER of 1.0088. The absolute disparity was similarly minimized in the BCL configuration (MMD: 0.0005) with high statistical significance. Notably, while TCL and SCL also demonstrated significant results over the baseline, they remained less effective than BCL in narrowing the performance range. The RI model again failed to provide a significant performance shift and showed poor subgroup consistency with a high MMD of 0.0300. While other approaches, including Self-Paced Curriculum Learning (SCL) and Teacher-Student Curriculum Learning (TCL), improved both fairness and accuracy relative to the Baseline model, they did not surpass the overall effectiveness of the BCL method.

These findings highlight the success of the BCL approach in balancing accuracy with fairness by neutralizing both absolute disparity and relative error gaps. In the following section, we explore how various loss functions affect segmentation performance to further enhance the model's results.

### 3.3 Progressive loss and CL

In this section, we further assess the performance of U-Net, our selected baseline model for segmentation, by examining the effects of Balanced Curriculum Learning (BCL) combined with progressive loss functions, Selective Progressive Loss (SPL) and Tiered Progressive Loss (TPL). The outcomes for both hip and knee segmentation are detailed in Tables 8, 9. To isolate the impact of the loss formulations in this section, statistical significance was assessed by comparing TPL configurations against their corresponding SPL counterparts (e.g., SPL-noBCL vs. TPL-noBCL and SPL-BCL vs. TPL-BCL).

**Table 8:** Progressive loss and hip segmentation with statistical and disparity metrics.

Model	Mean IoU	IoU SD	R&G mean SER	MMD	P-IoU
SPL - noBCL	0.9385	**0.0063**	1.0506	0.0152	–
SPL - BCL	0.9003	0.0125	1.1460	0.0329	–
TPL - noBCL	0.9476	**0.0063**	1.0549	**0.0151**	**<0.05**
TPL - BCL	**0.9532**	0.0125	**1.0129**	0.0255	**<0.05**

The bold values indicate the best-performing results for each metric. Specifically, higher values are highlighted for performance metrics such as IoU, while lower values are highlighted for disparity-related metrics like as SER and Min-Max Disparity.

**Table 9:** Progressive loss and knee segmentation with statistical and disparity metrics.

Model	Mean IoU	IoU SD	R&G mean SER	MMD	P-IoU
SPL - noBCL	0.9178	**0.0059**	1.0153	**0.0130**	–
SPL - BCL	0.9098	0.0104	1.0929	0.0269	–
TPL - noBCL	0.9270	**0.0059**	1.0214	0.0132	**<0.05**
TPL - BCL	**0.9668**	0.0104	**1.0121**	0.0212	**<0.05**

The bold values indicate the best-performing results for each metric. Specifically, higher values are highlighted for performance metrics such as IoU, while lower values are highlighted for disparity-related metrics like as SER and Min-Max Disparity.

For SPL, models trained without BCL generally outperformed those trained with BCL. Specifically, the SPL-noBCL setup achieved IoU scores of 0.9385 for hip and 0.9178 for knee, along with lower mean SER values, indicating improved fairness. This setup also maintained relatively low absolute performance gaps, with an MMD of 0.0152 and 0.0130 across the datasets. Conversely, adding BCL to SPL resulted in decreased performance; the SPL-BCL configuration showed reduced IoU scores (0.9003 for hip and 0.9098 for knee) and increased mean SER values. This suggests that the loss modulation in SPL may interfere with BCL's sample selection strategy, diminishing its effectiveness.

On the other hand, TPL consistently surpassed SPL on both datasets. The TPL-BCL combination delivered the highest IoU scores (0.9532 for hip and 0.9668 for knee) alongside the lowest mean SER values, demonstrating a strong balance between accuracy and fairness. When compared to the SPL-BCL configuration, the improvements in mean IoU and fairness for TPL-BCL were found to be highly significant, indicating that TPL's tiered approach better handles the complexity introduced by the curriculum. Furthermore, TPL-BCL significantly reduced the absolute MMD for both hip and knee tasks. This success is likely due to TPL's emphasis on challenging samples during the later phases of training. Remarkably, TPL also maintained solid performance without BCL, achieving IoU scores of 0.9476 for hip and 0.9270 for knee, highlighting its intrinsic adaptability and providing a statistically significant enhancement over the SPL-noBCL baseline while achieving lower disparity values (MMD).

However, comparing the results from Tables 6, 7 with those in Tables 8, 9, it is evident that the BCL strategy by itself outperforms all progressive loss methods across nearly all evaluation metrics, except for the mean IoU in knee segmentation. This comparison reinforces the effectiveness of a difficulty-based curriculum over loss weighting alone in minimizing both relative bias (SER) and absolute performance ranges (MMD) across subgroups.

### 3.4 Collection sites: data distribution

To simulate real-world federated learning scenarios, we partitioned both the hip and knee datasets according to the clinical acquisition site, represented by the variable V00SITE. Each site corresponds to a distinct medical center where samples were collected. In FL settings, such partitioning reflects a decentralized training environment, where data remains locally stored and model updates are shared instead of raw patient data.

This site-based partitioning introduces significant heterogeneity across clients due to demographic imbalance at each location. Variations in gender, race, and intersectional subgroups (e.g., race-gender combinations) are evident and critical to understanding both performance and fairness in federated models. Tables 10, 11 present the demographic distributions across the five sites (A–E) in the hip and knee datasets, respectively.

**Table 10:** Demographic distribution by acquisition site in the hip dataset.

Attribute	Site A	Site B	Site C	Site D	Site E
Male	73	49	99	48	55
Female	140	80	50	70	97
White or Caucasian	56	124	131	82	50
Black or AA^*^	157	5	18	36	102
Male-Black (MB)	45	3	8	11	27
Male-White (MW)	28	46	91	37	28
Female-Black (FB)	112	2	10	25	75
Female-White (FW)	28	78	40	45	22

AA^*^, African American.

**Table 11:** Demographic distribution by acquisition site in the knee dataset.

Attribute	Site A	Site B	Site C	Site D	Site E
Male	66	38	60	78	38
Female	109	71	94	91	62
White or Caucasian	52	103	129	124	39
Black or AA^*^	123	6	25	45	61
Male-Black (MB)	43	2	4	14	23
Male-White (MW)	23	36	56	64	15
Female-Black (FB)	80	4	21	31	38
Female-White (FW)	29	67	73	60	24

AA^*^, African American.

As shown in Table 10, Site A in the hip dataset has a large proportion of Black individuals (157 out of 213), with 112 of them being Female-Black (FB), while Sites B and C are dominated by White participants. Site D and E show more balanced but still skewed distributions. A similar trend is observed in the knee dataset (Table 11), where Site A is again heavily skewed toward Black and FB participants, whereas Site B is largely White and Female-White (FW). This uneven distribution across sites highlights the Non-Independent and Identically Distributed (non-IID) nature of the data, underscoring the importance of fairness-aware training strategies.

### 3.5 General FL training approach

We begin by evaluating the performance of several standard federated learning algorithms when enhanced with Balanced Curriculum Learning (BCL) and Tiered Progressive Loss (TPL). These general approaches serve as a baseline for comparison against our proposed methods. The results for these algorithms are detailed in Table 12 for hip segmentation and Table 13 for knee segmentation. To ensure a fair evaluation across decentralized frameworks, statistical significance was determined by using the standard FedAvg algorithm as the reference baseline. Specifically, models using Cross-Entropy (CE) were compared against FedAvg+CE, while TPL and BCL-enhanced models were compared against their matched FedAvg counterparts (e.g., FedDP+TPL vs. FedAvg+TPL) to isolate the effect of the aggregation algorithm.

**Table 12:** Federated learning performance (FedAvg, FedDP, FedProx) for hip segmentation.

Model	Mean IoU	IoU SD	R&G mean SER	MMD	P-IoU
FedAvg + CE	0.8976	0.0045	1.0948	0.0093	–
FedAvg + TPL	0.8943	0.0024	1.0317	0.0061	–
FedAvg + CE & BCL	0.9718	0.0003	1.0199	0.0008	–
FedAvg + TPL & BCL	**0.9740**	**0.0002**	**1.0155**	**0.0004**	–
FedDP + CE	0.9128	0.0022	1.0598	0.0051	**<0.05**
FedDP + TPL	0.8926	0.0039	1.0793	0.0082	**<0.05**
FedDP + CE & BCL	0.9128	0.0022	1.0598	0.0051	**<0.05**
FedDP + TPL & BCL	0.8956	0.0018	1.0400	0.0041	**<0.05**
FedProx + CE	0.9456	0.0008	1.0251	0.0020	**<0.05**
FedProx + TPL	0.9475	0.0003	1.0163	0.0009	**<0.05**
FedProx + CE & BCL	0.9589	0.0003	1.0196	0.0008	**<0.05**
FedProx + TPL & BCL	0.9723	**0.0002**	1.0164	0.0005	**<0.05**

The bold values indicate the best-performing results for each metric. Specifically, higher values are highlighted for performance metrics such as IoU, while lower values are highlighted for disparity-related metrics like as SER and Min-Max Disparity.

**Table 13:** Federated learning performance (FedAvg, FedDP, FedProx) for knee segmentation.

Model	Mean IoU	IoU SD	R&G mean SER	MMD	P-IoU
FedAvg + CE	0.9118	0.0034	1.0569	0.0086	–
FedAvg + TPL	0.9104	0.0020	1.0309	0.0052	–
FedAvg + CE & BCL	0.9104	0.0020	1.0309	0.0052	–
FedAvg + TPL & BCL	0.9483	0.0005	1.0216	0.0011	–
FedDP + CE	0.9101	0.0024	1.0600	0.0053	**<0.05**
FedDP + TPL	0.9018	0.0021	1.0393	0.0053	**<0.05**
FedDP + CE & BCL	0.9207	0.0013	1.0274	0.0035	**<0.05**
FedDP + TPL & BCL	0.9033	0.0038	1.0772	0.0091	**<0.05**
FedProx + CE	0.9616	0.0007	1.0320	0.0019	**<0.05**
FedProx + TPL	0.9615	**0.0003**	**1.0184**	**0.0007**	**<0.05**
FedProx + CE & BCL	**0.9617**	**0.0003**	1.0199	0.0008	**<0.05**
FedProx + TPL & BCL	0.9516	0.0005	1.0188	0.0013	**<0.05**

The bold values indicate the best-performing results for each metric. Specifically, higher values are highlighted for performance metrics such as IoU, while lower values are highlighted for disparity-related metrics like as SER and Min-Max Disparity.

For FedAvg, the standard algorithm using Cross-Entropy (CE) loss established a baseline performance for hip segmentation with a mean IoU of 0.8976. The combination of FedAvg with both BCL and TPL enhanced performance, achieving a mean IoU of 0.9740 and an SER of 1.0155. This configuration also substantially reduced the absolute performance gap, yielding the lowest Min-Max Disparity (MMD) of 0.0004 among the baseline aggregation methods. On the knee data, integrating both BCL and TPL with FedAvg also substantially improved metrics from a mean IoU of 0.9118 to 0.9483. This demonstrates that even within a basic FL framework, these local strategies can synergistically improve both accuracy and fairness.

Regarding FedDP, which incorporates client-level differential privacy, the hip model with CE achieved a mean IoU of 0.9128. However, when both BCL and TPL were added, the mean IoU decreased to 0.8956. A similar trend was observed for knee segmentation, where the full combination with local enhancements resulted in a mean IoU of 0.9033 and an SER of 1.0772. Statistical analysis revealed that the performance shifts in FedDP configurations were significant compared to the FedAvg baseline, highlighting a distinct privacy-utility trade-off. The introduction of Gaussian noise appears to counteract the stability gains provided by BCL, often resulting in higher MMD values compared to non-private counterparts. This suggests a potential trade-off when combining these advanced local training methods with the noise addition inherent in this FedDP implementation, which may require further tuning.

In the case of FedProx, designed to handle statistical heterogeneity, the CE configuration for hip segmentation showed strong initial performance. The full combination yielded one of the top-tier results: a mean IoU of 0.9723 and an SER of 1.0164, showcasing FedProx's robustness. FedProx consistently demonstrated statistically significant improvements in stability over FedAvg, also reflected in achieving an MMD as low as 0.0005 for hip segmentation. For knee segmentation, the model with TPL was highly effective on its own, achieving a mean IoU of 0.9615. The addition of BCL resulted in a slightly lower mean IoU of 0.9516 but maintained a comparable SER, indicating strong performance across both datasets with statistically significant gains over standard FedAvg baselines.

### 3.6 Fairness-focused FL training approach

Next, we evaluate our novel algorithms that incorporate fairness-aware mechanisms directly into the federated aggregation process. The performance of these fairness-focused approaches is presented in Table 14 for hip segmentation and Table 15 for knee segmentation. To establish the relative advantage of these fairness-aware mechanisms, statistical significance was assessed by comparing each configuration against its corresponding setup in the standard FedDPfair algorithm. This comparison accounts for the privacy-utility trade-offs inherent in distributed clinical data.

**Table 14:** Federated learning performance (FedDPfair, FedIoU, FedIoUoutlier) for hip segmentation.

Model	Mean IoU	IoU SD	R&G mean SER	MMD	P-IoU
FedDPfair + CE	0.8918	0.0050	1.0691	0.0136	–
FedDPfair + TPL	0.9028	0.0026	1.0529	0.0061	–
FedDPfair + CE & BCL	0.9189	0.0013	1.0504	0.0040	–
FedDPfair + TPL & BCL	0.9375	0.0012	1.0451	0.0028	–
FedIoU + CE	0.9567	0.0006	1.0269	0.0014	**<0.05**
FedIoU + TPL	0.9581	0.0003	1.0156	0.0007	**<0.05**
FedIoU + CE & BCL	0.9689	0.0003	1.0178	0.0007	**<0.05**
FedIoU + TPL & BCL	0.9744	**0.0002**	1.0138	0.0005	**<0.05**
FedIoUoutlier + CE	0.9628	0.0004	1.0190	0.0011	**<0.05**
FedIoUoutlier + TPL	0.9701	0.0003	1.0186	0.0008	**<0.05**
FedIoUoutlier + CE & BCL	0.9729	**0.0002**	1.0149	**0.0004**	**<0.05**
FedIoUoutlier + TPL & BCL	**0.9756**	**0.0002**	**1.0124**	0.0005	**<0.05**

The bold values indicate the best-performing results for each metric. Specifically, higher values are highlighted for performance metrics such as IoU, while lower values are highlighted for disparity-related metrics like as SER and Min-Max Disparity.

**Table 15:** Federated learning performance (FedDPfair, FedIoU, FedIoUoutlier) for knee segmentation.

Model	Mean IoU	IoU SD	R&G mean SER	MMD	P-IoU
FedDPfair + CE	0.9235	0.0047	1.1164	0.0126	–
FedDPfair + TPL	0.9077	0.0048	1.1097	0.0097	–
FedDPfair + CE & BCL	0.9358	0.0011	1.0347	0.0024	–
FedDPfair + TPL & BCL	0.9370	0.0008	1.0284	0.0018	–
FedIoU + CE	0.9468	0.0007	1.0198	0.0019	**<0.05**
FedIoU + TPL	0.9617	0.0003	1.0171	0.0007	**<0.05**
FedIoU + CE & BCL	0.9612	0.0004	1.0183	0.0011	**<0.05**
FedIoU + TPL & BCL	0.9628	0.0002	1.0164	0.0006	**<0.05**
FedIoUoutlier + CE	0.9619	0.0003	1.0173	0.0007	**<0.05**
FedIoUoutlier + TPL	0.9638	0.0003	1.0168	0.0006	**<0.05**
FedIoUoutlier + CE & BCL	0.9719	0.0002	1.0144	0.0004	**<0.05**
FedIoUoutlier + TPL & BCL	**0.9734**	**0.0001**	**1.0114**	**0.0003**	**<0.05**

The bold values indicate the best-performing results for each metric. Specifically, higher values are highlighted for performance metrics such as IoU, while lower values are highlighted for disparity-related metrics like as SER and Min-Max Disparity.

Considering FedDPfair, an algorithm that weights client updates by the inverse of their SER (1/SER), it exhibited moderate performance. For hip segmentation, the combination with BCL and TPL improved figures to a mean IoU of 0.9375 and an SER of 1.0451. For the knee dataset, this combination achieved a mean IoU of 0.9370 and an SER of 1.0284, a notable improvement over its CE baseline, with MMD showing a consistent and similar trend. This indicates that while the fairness-aware aggregation helps, the overall performance benefits substantially from the inclusion of BCL and TPL.

For FedIoU and FedIoUoutlier, that weight client contributions by their locally achieved IoU scores, superior performance was demonstrated. For hip segmentation, FedIoU combined with BCL and TPL achieved a high mean IoU of 0.9744. This setup yielded highly significant performance gains compared to the FedDPfair baseline, effectively narrowing the absolute performance range to an MMD of 0.0005. The FedIoUoutlier variant, which further refines aggregation by excluding statistical outliers, excelled, delivering the highest mean IoU at 0.9756 and an SER of 1.0124. Crucially, FedIoUoutlier combined with CE and BCL achieved the absolute minimum absolute gap with an MMD of 0.0004. These IoU-weighted algorithms again led the performance for knee segmentation. The top-performing configuration is FedIoUoutlier with BCL and TPL, which yielded the highest mean IoU of 0.9734, the lowest IoU SD of 0.0001, and an SER of 1.0114 with an unmatched absolute MMD of 0.0003.

The federated learning experiments consistently demonstrate that incorporating local training enhancements like BCL and TPL significantly boosts the performance of various FL algorithms in terms of both accuracy (mean IoU) and fairness (mean SER). While baseline FedAvg benefits considerably from these local strategies, more sophisticated FL algorithms such as FedProx, FedIoU, and particularly FedIoUoutlier, show even greater potential when combined with these methods. The consistently low MMD values across fairness-focused aggregations further support the fairness trends observed with SER, indicating limited demographic subgroup disparities. However, these metrics primarily reflect subgroup-level fairness and should not be interpreted as definitive evidence against potential majority-site bias in IoU-based aggregation.

The FedIoUoutlier algorithm, when coupled with both BCL and TPL for local client training, emerged as the most effective FL strategy across both hip and knee segmentation tasks. Its mechanism of weighting client updates by their local IoU scores and excluding statistical outliers from the aggregation process appears to interact synergistically with local curricula that manage sample difficulty (BCL) and loss functions that adaptively focus on challenging instances (TPL), suggesting that stronger and more stable local training improves the quality of performance-aware global aggregation. This combination consistently yielded the highest mean IoU scores and among the best mean SER values across both datasets, along with minimal variance (IoU SD), and absolute performance gap (MMD). Moreover, although outlier updates may be excluded in a given round, subsequent global updates still provide those clients with a stronger shared model initialization in later rounds, allowing them to continue benefiting from collective learning rather than being permanently marginalized. At the same time, although IoU-based weighting improved aggregate performance and subgroup fairness metrics in our experiments, such weighting may preferentially amplify updates from stronger-performing clients, and therefore its implications for site-level balance warrant further investigation. Taken together, these FL results illustrate several important trade-offs. First, privacy-enhanced aggregation through FedDP and FedDPfair can preserve confidentiality but may reduce segmentation quality when noise disrupts locally learned representations. Second, heterogeneity across sites can introduce instability and client drift, making aggregation quality especially important under non-IID conditions. Third, aggregation rules themselves may influence fairness: sample-size weighting can favor larger clients, whereas IoU-based weighting can favor stronger-performing clients. Within this context, the combination of BCL, TPL, and FedIoU-based aggregation appears to improve both local model quality and global update selection, helping to balance utility and subgroup fairness more effectively than standard FL baselines.

## 4 Discussion

This study demonstrates that Curriculum Learning (CL), Progressive Loss (PL), and Federated algorithms with fairness (FedIoU and FedIoUoutlier) can jointly improve segmentation performance while reducing disparities across demographic subgroups. The consistent reduction in standard deviation of IoUs, relative error ratios (SER), and absolute performance gaps (MMD) across centralized and distributed experiments supports the robustness of the proposed approach. These gains were assessed through paired *t*-tests, showing that the incorporation of Balanced Curriculum Learning (BCL) and Tiered Progressive Loss (TPL) yielded statistically significant improvements relative to their matched comparison configurations across both centralized and federated settings. These findings highlight the importance of embedding fairness objectives directly into model training rather than relying solely on *post-hoc* evaluation.

Importantly, the federated results should be interpreted through the lens of FL-specific trade-offs. While distributed training preserves data privacy, it also introduces communication overhead, sensitivity to non-IID client distributions, and the possibility of client drift across rounds. Our results suggest that these factors are not merely engineering concerns, but can materially influence both segmentation accuracy and subgroup fairness. In particular, the weaker performance of privacy-enhanced FedDP variants reflects the privacy–utility tension, whereas the stronger performance of FedIoU and FedIoUoutlier indicates that more selective aggregation can partially offset instability introduced by heterogeneous client updates. The combined benefit of CL, PL, and FedIoU-based aggregation also appears to be mechanistically coherent. BCL improves the composition and progression of local training data, TPL progressively emphasizes harder cases during optimization, and FedIoU-based aggregation selectively amplifies clients that convert these local advantages into higher-quality segmentation updates. Rather than acting independently, the three components operate at complementary stages of training and aggregation, which likely explains why their combination consistently produced the strongest federated results.

From a health equity perspective, improving segmentation consistency across populations may help reduce downstream disparities in musculoskeletal imaging, surgical planning, and disease monitoring. The federated framework further shows promise for deployment in multi-institutional collaborations where data sharing is restricted, enabling collective improvements while preserving patient privacy. The strong performance of FedIoUoutlier in mitigating outlier-driven instability suggests that high-fidelity models can still be collaboratively learned despite noisy or heterogeneous site-specific data distributions. In addition, exclusion as an outlier in a given round does not imply permanent omission, since subsequent rounds still provide clients with an improved global model initialization for continued local training. Beyond radiography, the methodological framework outlined here has potential applicability to other imaging modalities and clinical domains where inequities and institutional silos remain persistent challenges.

At the same time, several limitations should be acknowledged. The demographic analysis was limited to Black and White subgroups, constraining the generalizability of fairness findings to more diverse populations. The evaluation focused on a single dataset of hip and knee radiographs, and external validation across different imaging modalities and healthcare systems will be necessary to confirm robustness. In addition, federated training introduces communication and computational overhead that may pose challenges in resource-constrained environments. Although the publicly available OAI dataset provides a valuable basis for fairness analysis and includes demographic metadata such as race and gender, it remains a single coordinated cohort and therefore does not fully capture the broader variability that may arise across independently governed hospitals, including differences in imaging workflows, scanner characteristics, clinical populations, and institutional practices. In addition, while fairness was evaluated using SER and MMD to enable compact and consistent comparison across multiple centralized and federated configurations, these summary measures do not fully characterize all subgroup- and site-specific behaviors. More detailed subgroup and client-level reporting would further strengthen the fairness assessment. Another limitation is that statistical significance testing was conducted on per-sample IoU scores, whereas subgroup-level fairness metrics were interpreted primarily as consistent empirical trends. Furthermore, weighting strategies in federated learning may inherently favor certain clients: standard aggregation can emphasize clients with larger local sample sizes, while IoU-based weighting may also favor clients with stronger local performance. Thus, although subgroup fairness metrics remained stable, potential site-level imbalance cannot be fully ruled out. Future work should also quantify communication costs and convergence behavior more explicitly, particularly under stronger client heterogeneity, to better characterize the practical deployment trade-offs of fairness-aware federated segmentation.

## 5 Conclusion

This study aimed to enhance both the accuracy and fairness of medical image segmentation for hip and knee radiographs, addressing the critical challenge of demographic biases in AI-driven healthcare. Our investigation, leveraging a comprehensive dataset from the Osteoarthritis Initiative, explored the efficacy of various model architectures, Curriculum Learning (CL) techniques, and Progressive Loss (PL) functions within both centralized and, crucially, privacy-preserving federated learning frameworks.

Initial evaluations in a centralized setting underscored that while baseline models like U-Net can achieve high segmentation accuracy, they may still exhibit demographic biases, necessitating targeted interventions. The application of CL strategies, particularly Balanced Curriculum Learning (BCL), demonstrated substantial improvements, yielding higher IoU scores and lower Skewed Error Ratios (SER), thereby enhancing both accuracy and fairness. The very small absolute gaps achieved through BCL, as measured by min-max disparity (MMD), further highlight its effectiveness in promoting demographic equity. The integration of Tiered Progressive Loss (TPL) with BCL further refined these results in certain centralized configurations.

Significantly, our exploration extended these fairness-enhancing strategies to a federated learning environment, simulating real-world scenarios where data is distributed and privacy is paramount. This evaluation also benefits from the use of the publicly available OAI cohort, a multicenter study with data collected across multiple clinical sites, which provides a meaningful testbed for studying federated learning under geographically distributed acquisition settings. The results from the FL analysis were particularly compelling. We found that advanced FL algorithms, when combined with local training strategies incorporating BCL and TPL, can achieve remarkable performance. Specifically, configurations such as FedIoUoutlier, which intelligently manages contributions from participating clients by statistically pruning anomalous updates, when paired with BCL and TPL for local model training, yielded state-of-the-art results. Remarkably, these FL setups matched or, in several instances, surpassed the accuracy and fairness metrics of the optimized centralized models with strong statistical significance. More broadly, the study shows that local curriculum design, adaptive loss shaping, and fairness-aware aggregation are most effective when treated as complementary components of the federated learning pipeline rather than as isolated interventions.

Ultimately, this research contributes advanced methodologies for building more accurate, fair, and privacy-respecting AI models for medical image segmentation. By demonstrating the synergistic benefits of CL, PL, and advanced FL algorithms, we pave the way for the development and deployment of AI tools that can help reduce healthcare disparities and ensure equitable outcomes across diverse demographic groups in distributed healthcare ecosystems.

## Data Availability

Publicly available datasets were analyzed in this study. This data can be found here: https://www.niams.nih.gov/grants-funding/funded-research/osteoarthritis-initiative.
